# Methylation of *Wnt7a* Is Modulated by DNMT1 and Cigarette Smoke Condensate in Non-Small Cell Lung Cancer

**DOI:** 10.1371/journal.pone.0032921

**Published:** 2012-03-05

**Authors:** Meredith A. Tennis, Michelle M. VanScoyk, Lora A. Wilson, Nicole Kelley, Robert A. Winn

**Affiliations:** Department of Medicine, University of Colorado School of Medicine, Aurora, Colorado, United States of America; Virginia Commonwealth University, United States of America

## Abstract

Wnt7a is known to be a tumor suppressor that is lost in NSCLC, but no mechanism of loss has been established. Methylation of promoter regions has been established as a common mechanism of loss of tumor suppressor expression in NSCLC. We previously demonstrated that loss of Wnt7a in non-transformed lung epithelial cell lines led to increased cell growth, altered 3-D culture growth, and increased migration. The *Wnt7a* promoter has a higher percentage of methylation in NSCLC tumor tissue compared to matched normal lung tissue and methylation of the promoter region leads to decreased activity. We treated H157 and H1299 NSCLC cell lines with 5-Aza-2′-deoxycytidine and detected loss of *Wnt7a* promoter methylation, increased Wnt7a expression, and increased activity of the Wnt7a lung signaling pathway. When DNMT1 expression was knocked down by shRNA, expression of Wnt7a increased and methylation decreased. Together these data suggest that in NSCLC, Wnt7a is lost by methylation in a subset of tumors and that this methylation is maintained by DNMT1. Restoration of Wnt7a expression through demethylation could be an important therapeutic approach in the treatment of NSCLC.

## Introduction

Lung cancer continues to be the leading cause of cancer death, with minimal options available for treatment of tumors typically diagnosed at late stages. We are interested in the role of non-canonical Wnt signaling in non-small lung cancer (NSCLC), specifically the tumor suppressive activities of Wnt7a and its receptor Frizzled 9 (Fzd9). Wnt7a is highly expressed in embryonic lung and serves to maintain a normal epithelial phenotype in the adult lung [1], [2]. Previous studies have demonstrated that Wnt7a is frequently lost in NSCLC and that restoration of Wnt7a expression leads to decreased transformed growth in NSCLC cell lines [2]. NSCLC cells with reexpression of Wnt7a have decreased proliferation, decreased anchorage independent growth, and restoration of an epithelial phenotype [2]. Wnt7a is located on chromosome 3p25, which is a “hotspot” for deletion, however, we suspected that Wnt7a expression in NSCLC can be regulated by an epigenetic mechanism [3].

Methylation of tumor suppressor gene promoters in NSCLC has been reported in over 40 genes, some with frequencies as high as 100% [4]. Loss of expression of genes such as p16/INK4a by methylation early in the development of NSCLC has been proposed as a biomarker for early detection [5]. Methylation of genes such as FHIT have been correlated with tumor staging and prognosis in NSCLC [6]. Differences in methylation associated with tumor histology have been identified for genes such as RASSF1A and p16/INK4a [7], [8]. Associations between exposure to cigarette smoke and aberrant methylation have been observed for p16, RASSF1A, and RARβ2 among others [9], [10], [11]. A link between exposure to tobacco carcinogens and methylation may exist through DNA Methyltransferases (DNMT) that are activated by these carcinogens. If targets of DNMTs responsible for DNA repair become inappropriately inactivated by methylation, lesions resulting from carcinogens may persist, leading to tumorigenesis [12].

As most Wnt and Wnt-related protein studies in cancer are related to canonical signaling, the identification of Wnt-specific methylation is not common. Methylation is known to be a mechanism of loss for other tumor suppressors in NSCLC, but most studies of Wnt methylation in the lung target Wnt antagonists in the canonical Wnt signaling pathway. A few studies have identified hypermethylation of Wnt5a and Wnt9a in epithelial cancers and hypermethylation of Wnt7a has been observed in a subset of ductal pancreatic cancer [13], [14], [15], [16]. In the present study, we have demonstrated that loss of Wnt7a is a significant event for lung epithelial cells and that this loss is caused by methylation through DNMT1 activity in a subset of NSCLC.

## Results

### Loss of Wnt7a expression in lung cells leads to a transformed phenotype

**Figure 1:**
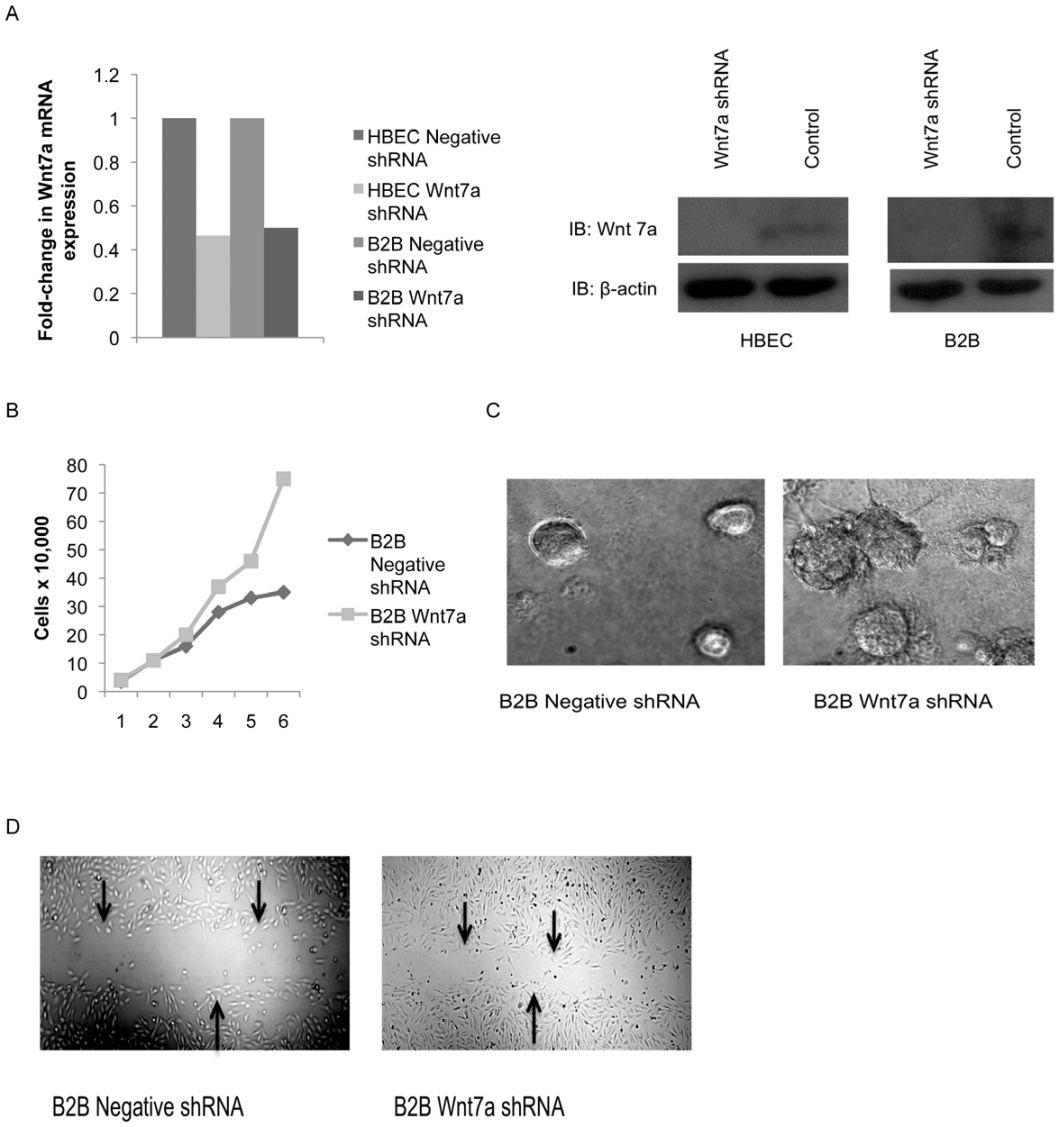
Effects of Wnt7a loss in lung epithelial cells. A) Expression of Wnt7a was measured by QPCR in HBEC and B2B cells after stable transfection with Wnt7a shRNA or a negative shRNA. Data is presented as fold-change in Wnt7a expression normalized to GAPDH. Error bars are the SE of triplicate experiments. Wnt7a knock down was also confirmed by western blot with a β-actin loading control. B) Cell proliferation was measured in B2B cells expressing Wnt7a shRNA or a negative shRNA. Cells were analyzed in a 6-day growth assay. C) B2B cells with Wnt7a shRNA were analyzed in a 3D culture assay using Matrigel and observed after 5 days of growth for changes in morphology compared to a negative shRNA control. B2B cells with Wnt7a shRNA were also analyzed in a scratch migration assay and observed at 24 hours for movement into the introduced scratch compared to a negative shRNA control. Arrows indicate edges of the scratch. Pictures represent triplicate experiments.

We have previously demonstrated that Wnt7a expression alters the transformed characteristics of NSCLC cells. NSCLC cells with reintroduced Wnt7a have decreased proliferation, decreased anchorage independent growth, and restoration of an epithelial phenotype. The converse, loss of Wnt7a from a non-transformed epithelial cell, would be expected to result in an increase in the characteristics of transformed cells. In this study, we used an shRNA approach to knock down mRNA expression of Wnt7a in two non-transformed lung epithelial cell lines, HBEC and Beas2B (B2B), and confirmed knock down by QPCR and western blot (Fig. 1A). Decreased Wnt7a expression led to increased cell proliferation in B2B cells, demonstrated by a 6-day cell growth assay (Fig. 1B). HBEC cell proliferation was also increased with loss of Wnt7a as measured by MTS assay (data not shown). In B2B cells, altered growth in Matrigel 3D culture was observed at 5 days with loss of Wnt7a expression (Fig. 1C). Increased cell migration in a scratch assay at 24 hours was observed with loss of Wnt7a expression, where arrows indicated the edges of the introduced scratch (Fig. 1C).

### Wnt7a is methylated in human NSCLC cell lines and tissues

**Figure 2:**
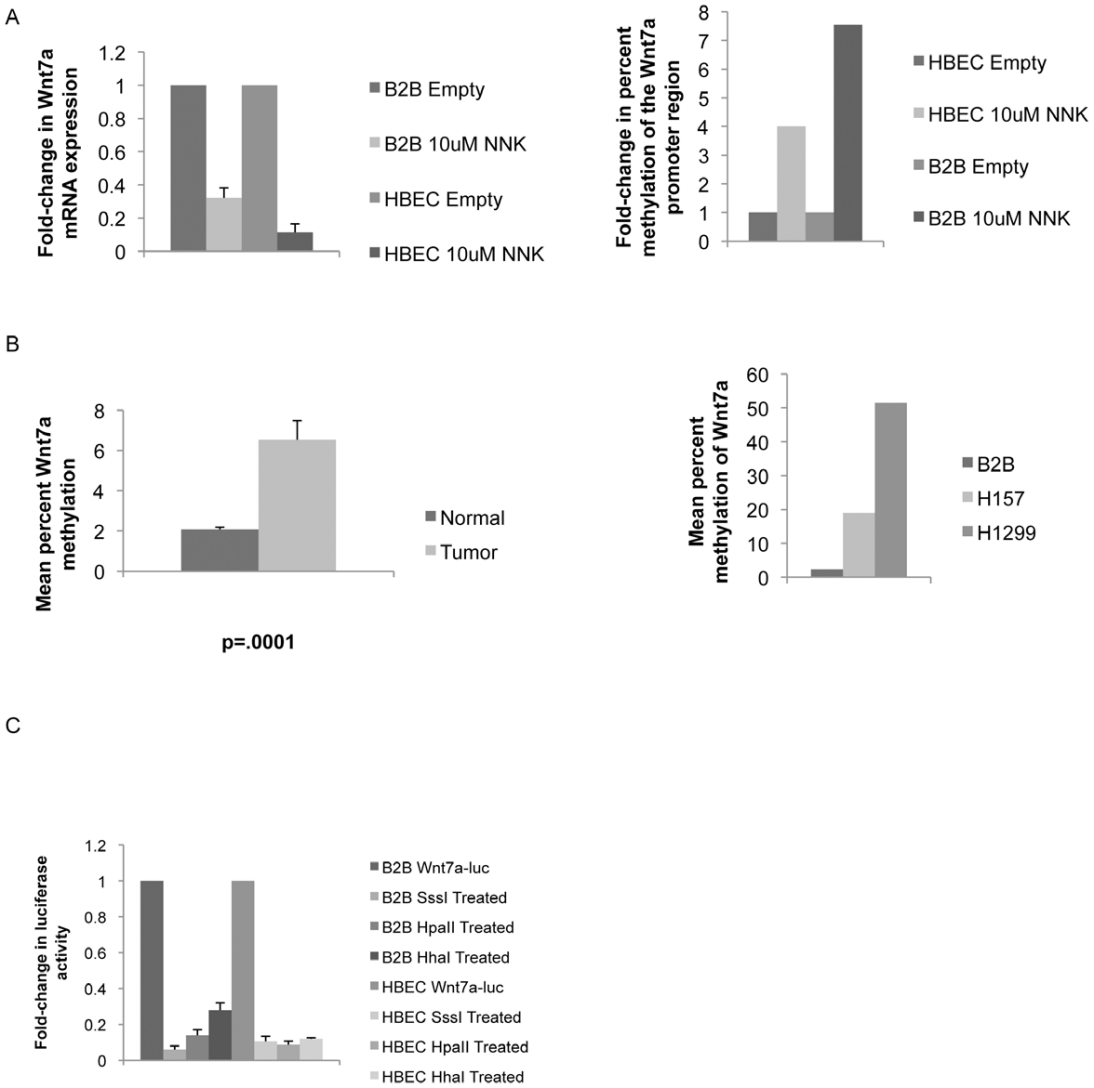
Wnt7a is methylated in NSCLC cell lines and tissues. A) B2B and HBEC cells were treated with 10 uM NNK for 2 hours and Wnt7a expression was measured by QPCR compared to an untreated control. Error bars are the SEM of triplicate experiments. Percent methylation in B2B and HBEC cells was analyzed using the Methyl Profiler system after 2 hr 10 uM NNK treatment compared to untreated cells. B) Mean percent methylation of the Wnt7a promoter was measured by pyrosequencing in NSCLC tissue and compared to matched normal adjacent lung tissue by paired t-test. Mean percent methylation of B2B, H157, and H1299 cell lines was measured by pyrosequencing. C) A Wnt7a promoter luciferase was modified by SssI, HpaII, and HhaI methylating enzymes and transiently transfected into B2B and HBEC cell lines. Fold-change in luciferase activity was measured compared to unmodified Wnt7a promoter luciferase activity. Error bars are the SE of triplicate experiments.

The question of how Wnt7a expression is lost could be addressed in several ways. *Wnt7a* could be deleted, mutated, or methylated, among other mechanisms. While the chromosome containing *Wnt7a* (3p25) is frequently deleted in NSCLC, this mechanism of loss, along with gene mutation, currently offers little in the way of therapeutic intervention [17]. The presence of the epigenetic mechanism of methylation would offer the possibility of using current clinical interventions to treat NSCLC. We treated HBEC and B2B cells with tobacco carcinogen NNK, which is known to lead to accumulation of DNMT1 and increased tumor suppressor hypermethylation in NSCLC [18]. In the treated cells we observed a decrease in Wnt7a mRNA expression by QPCR compared to untreated cells (Fig. 2A). Methylation analysis demonstrated that the Wnt7a promoter in B2B and HBEC cells has increased methylation after exposure to 10 uM NNK (Figure 2A). This suggested that *Wnt7a* is methylated with exposure to tobacco and may be a mechanism of loss in tobacco-associated NSCLC. In order to determine whether methylation of *Wnt7a* was present in human lung tumor tissue, we measured methylation of a CpG island in the promoter in 82 pairs of human lung tumor tissue and matched normal lung tissue. Mean percent methylation of the 13 CpG sites measured was significantly higher in tumor tissue compared to normal tissue when analyzed by a paired t-test (Fig. 2B). Table S1 shows percent methylation at each CpG site analyzed and compared between tumor and normal tissues. No single CpG site analyzed can be identified as critical to inactivation of the *Wnt7a* promoter, however, there appears to be a trend toward increased methylation in tumors at CpG sites closer to the transcription start site. Two cell NSCLC cell lines, H157 and H1299, were identified as harboring *Wnt7a* promoter methylation by pyrosequencing and were used for subsequent in vitro experiments (Fig. 2B). These data suggest that loss of Wnt7a expression by methylation occurs in a subset of NSCLC.

### The Wnt7a promoter deactivated by methylation is restored with 5-aza-2′ deoxycytidine treatment

**Figure 3:**
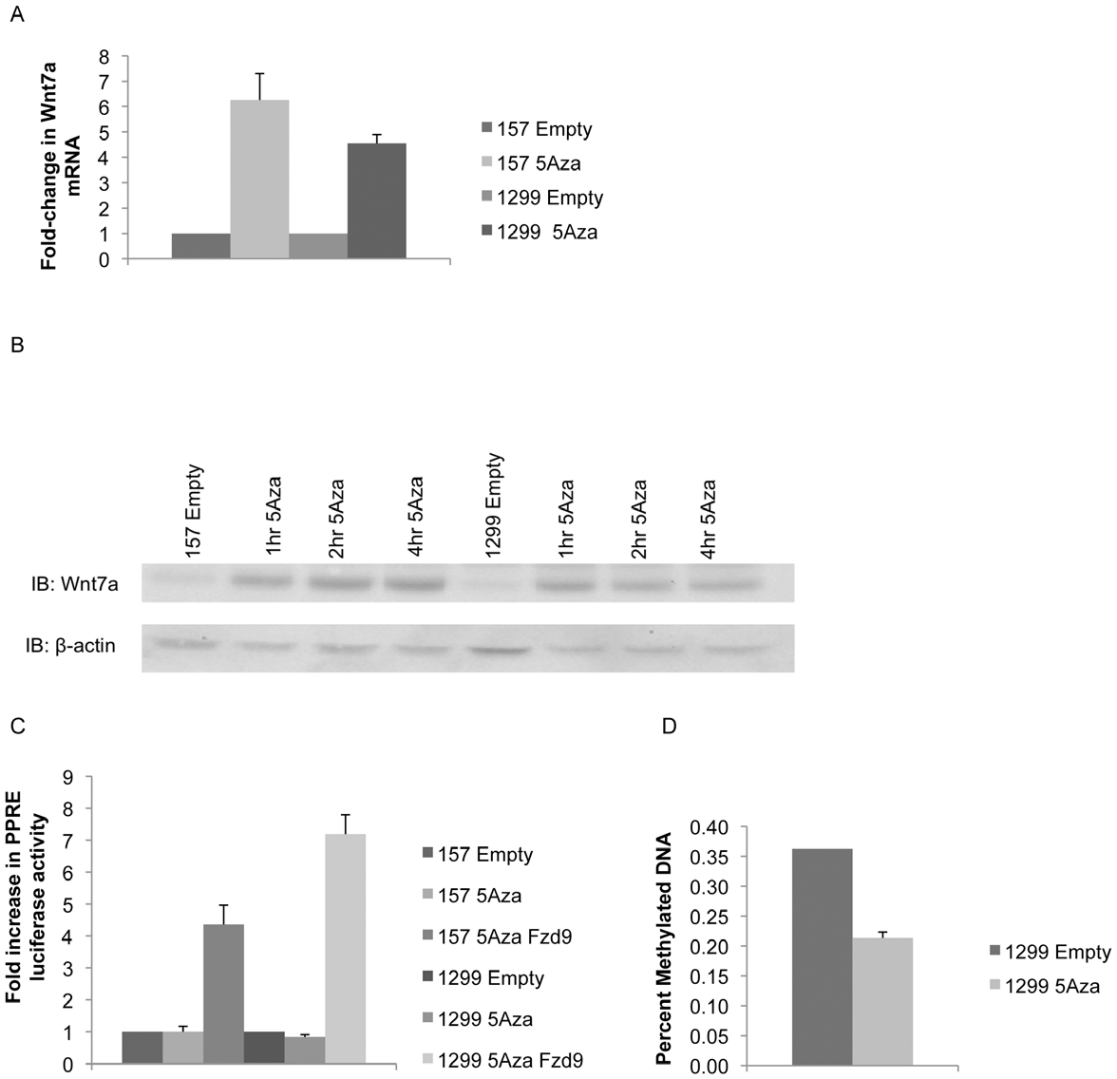
Treatment with 5 Aza reduces Wnt7a methylation. A) 157 and 1299 cells were treated with 5 Aza for 2 hours and Wnt7a was measured by QPCR compared to untreated controls. Error bars are the SEM of triplicate experiments. B) 157 and 1299 cells were treated with 5 Aza for 1, 2, and 4 hours and compared to untreated cells. Wnt7a protein expression was measured by immunoblot with β-actin as a loading control. C) 157 and 1299 cells were transiently transfected with Fzd9 and PPRE luciferase and 48 hours after were treated with 5 Aza for 2 hours. Luciferase activity was measured and treated cells were compared to an empty control. Error bars are the SE of triplicate experiments. D) 1299 cells were treated with 5 Aza for 2 hours and percent methylation of the Wnt7a promoter was measured by the Methyl-Profiler system. Treated cells were compared to an untreated control. Error bar is the SE of triplicate experiments.

As methylation of *Wnt7a* had not been studied previously, we wanted to verify that methylation of the promoter region would lead to inactivation of the promoter. A *Wnt7a* promoter luciferase was treated with SssI, HpaII, and HhaI methylases to determine the effect of methylation on the *Wnt7a* promoter region. SssI affects all CpG sites, HpaII only the CpG within 5″CCGG, and HhaI only the CpG within 5′GCGC. Transfection of the *Wnt7a* promoter luciferase into B2B and HBEC cell lines demonstrated decreased luciferase activity after treatment with all three methylases compared to an untreated *Wnt7a* promoter luciferase, indicating that even limited methylation inactivates the *Wnt7a* promoter (Fig. 2C). We had identified two NSCLC cell lines with methylation of the *Wnt7a* promoter region, H157 and H1299, and wanted to determine the effect of treatment with a demethylating agent, 5-aza-2′ deoxycytidine (5 Aza). Cells were treated with 6 uM 5 Aza for 1–4 hours and expression of Wnt7a was measured by QPCR and western blot. Exposure to 5 Aza led to increased expression of Wnt7a mRNA and protein (Fig. 3A and B). As expected, 5 Aza also led to decreased methylation of the *Wnt7a* promoter region (Fig. 3D). Wnt7a is known to signal through its receptor Fzd9 to downstream target PPARγ, so we treated H157 and H1299 cells with 5 Aza, along with transient transfection of Fzd9, and analyzed the activity of the Wnt7a signaling pathway [19]. The PPAR response element luciferase (PPRE), which is activated by PAPRγ, demonstrated increased activity after cells with methylated *Wnt7a* were exposed to 5 Aza and expression of Fzd9 (Fig. 3C). These data indicate that pharmaceutical treatment of NSCLC cells can reverse the methylated state of the *Wnt7a* promoter.

### Wnt7a promoter methylation is modulated by DNMT1

**Figure 4:**
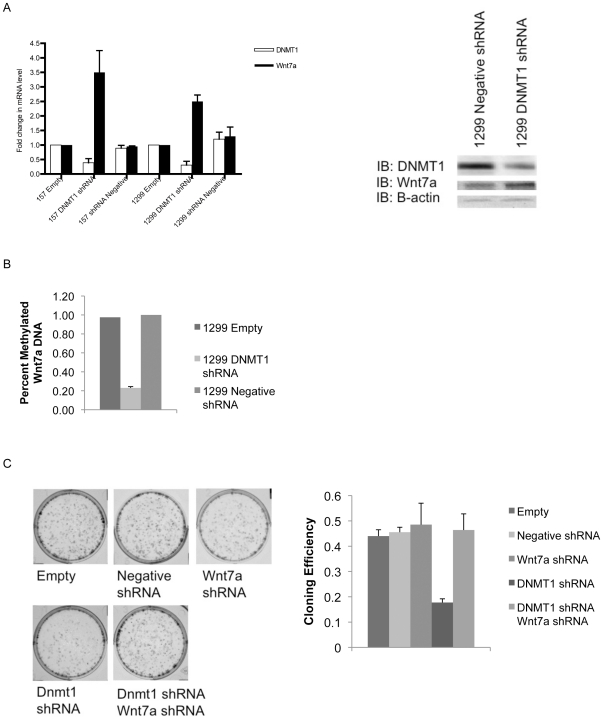
DNMT1 modulates Wnt7a promoter methylation. A) 1299 cells were transiently transfected with DNMT1 shRNA and compared to empty and shRNA negative control-transfected cells. DNMT1 and Wnt7a mRNA expression was measured by QPCR and presented as fold-change compared to the empty control. Error bars are the SE of triplicate experiments. DNMT1 shRNA and a shRNA negative control were stably transfected into 1299 cells and DNMT1 and Wnt7a protein were measured by immnoblot with a β-actin loading control. B) Transiently transfected 1299 cells were analyzed for Wnt7a promoter methylation using pyrosequencing. DNMT1 shRNA and a negative shrNA control transfected cells were compared to empty cells. Error bars are the SE of triplicate experiments. C) 1299 cells with transient DNMT1 shRNA and/or Wnt7a shRNA expression were compared to an empty control and negative shRNA control in a clonogenicity assay. Cells were stained after 4 days of growth, photographed, and counted for cloning efficiency. Error bars are the SE of duplicate experiments.

DNA methyltransferases (DNMT) modulate DNA methylation and their overexpression has been associated with lung cancer [4]. DNMT1 is known to accumulate in the nucleus of NSCLC cells in response to exposure to tobacco carcinogen NNK; we found that exposure to NNK decreased expression of Wnt7a mRNA measured by QPCR (Fig. 2A) [18]. When we knocked down expression of DNMT1 in Wnt7A methylated cell lines H157 and H1299 using shRNA, expression of DNMT1 decreased and expression of Wnt7a correspondingly increased by QPCR (Fig. 4A). Decreased DNMT1 protein expression and increased Wnt7a expression was also observed in H1299 cells (Fig. 4A). As expected, methylation of Wnt7a in the DNMT1 knock down H1299 cells decreased when measured by pyrosequencing (Fig. 4B). Similar to a study by Kassis et al. that found decreased proliferation with RNAi depletion of DNMT1, we observed a decrease in clonogenicity in H1299 cells with knock down of DNMT1 compared to empty and negative shRNA transfections (Fig. 4C). We also observed a return to the clonogenicity of the empty transfection with the addition of Wnt7a shRNA to the DNMT1 shRNA treated cells. This decreased clonogenicity observed with DNMT1 shRNA treatment mimics decreases in proliferation observed with restoration of Wnt7a expression in previous studies [2]. This, combined with the increased clonogenicity observed with the addition of Wnt7a shRNA, suggests that loss of Wnt7a expression could be in part responsible for the negative effects associated with DNMT1 overexpression in NSCLC [20]. Knock down of DNMT3a or 3b in the H1299 cells did not result in increased Wnt7a expression, suggesting that DNMT1 is responsible for *de novo* and maintenance methylation of the *Wnt7a* promoter (Figure S1).

### Cigarette smoke condensate exposure leads to Wnt7a promoter methylation

**Figure 5:**
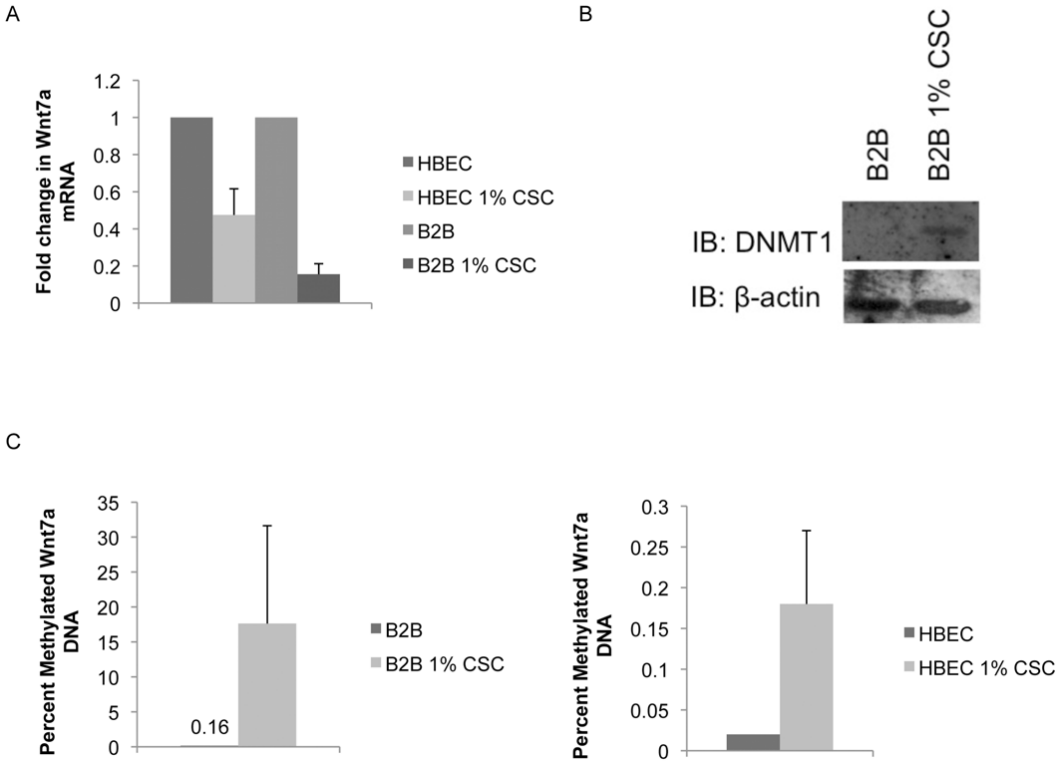
CSC treatment leads to Wnt7a methylation. A) HBEC and B2B cells were treated with 1% CSC in cell growth media for 12 weeks and Wnt7a expression was measured by QPCR. Error bars are the SE of triplicate experiments. B) Expression of DNMT1 was measured by western blot in B2B cells treated with 1% CSC for 12 weeks. Loading control is β-actin. C) HBEC and B2B cells treated with 1% CSC for 12 weeks were analyzed for Wnt7a promoter methylation compared to untreated controls using the Methyl-Profiler system. Error bars are the SE of triplicate experiments.

Methylation has been shown to be increased with exposure to tobacco carcinogens, so we were interested in the effect of cigarette smoke condensate (CSC) on methylation of the *Wnt7a* promoter [20]. We grew the non-transformed cell lines HBEC and B2B in 1% CSC media for 12 weeks and then extracted RNA, DNA, and protein. QPCR analysis found decreased expression of Wnt7a in both cell lines after 12 weeks of exposure to CSC (Fig. 5A). Increased DNMT1 protein expression was confirmed in B2B cells with CSC exposure (Fig. 5B). As expected, increased methylation of the *Wnt7a* promoter region was also identified in both cell lines (Fig. 5C). The exposure of lung epithelium to the carcinogens in cigarette smoke is associated with increased methylation and subsequent decreased expression of Wnt7a.

## Discussion

The data from this study support the role of Wnt7a as a tumor suppressor in lung epithelial cells and emphasize the role of Wnt7a in maintaining an epithelial phenotype in adult lung tissue. Wnt7a appears to be unique in the literature to date, as no other Wnt has been identified in NSCLC as a tumor suppressor or as relevant to the maintenance of a lung epithelial phenotype. Wnt5a is known to antagonize β-catenin signaling in esophageal cancer, but in NSCLC expression of Wnt5a appears to increase proliferation [13], [21], [22]. The significantly adverse effect of the loss of Wnt7a on lung epithelial cells motivated our determination of a mechanism of loss. Also important is the potential to address therapeutic strategies employing reexpression of Wnt7a.

Frequent loss of Wnt7a in NSCLC has been previously described and in the current study, we presented data supporting the importance of the expression of Wnt7a to the lung epithelium [2]. When Wnt7a expression is lost from non-transformed lung epithelial cell lines, the cells acquire characteristics of transformed cells and begin to more resemble NSCLC cell lines in proliferation rate, 3D culture growth, and migration. If this loss of Wnt7a were to occur in a human patient in combination with increased activity of oncogenes, the effects could lead to development of a lung tumor that has undergone EMT, which is associated with worse prognosis [23].

DNMT1 is thought to be responsible for maintaining methylation patterns and its elevated expression has been identified as a prognostic factor in NSCLC progression [24]. We found that when DNMT1 expression is reduced, Wnt7a methylation is reduced and expression increases. We have also shown that Wnt7a is responsible for a part of the decreased cell growth observed with DNMT1 loss. Typically, an additional DNMT is involved in specific de novo gene promoter methylation, while DNMT1 maintains this methylation. However, recent studies have reported that DNMT1 also has *de novo* methyltransferase activity in CpG regions [12]. Our finding that loss of DNMT3a or 3b expression does not result in increased Wnt7a expression in H1299 cells suggests that DNMT1 may be the sole DNMT responsible for promoter methylation of Wnt7a. If so, this may make therapeutic demethylation of *Wnt7a* through reduction of DNMT activity less complicated as only one DNMT would have to be targeted and monitored.

Exposure to tobacco carcinogens in vitro and in vivo has been associated with changes in DNMT activity and epigenetic alterations [11], [18], [25], [26]. In our study, treatment of non-transformed lung epithelial cells with CSC led to increased methylation of Wnt7a and a corresponding decrease in expression. A previous study by Liu et al. found decreased Wnt7a expression by array after CSC exposure [11]. Several studies have demonstrated increased DNMT1 protein expression or accumulation and increased methylation of specific genes after exposure to tobacco carcinogens [18], [25], [26]. Loss of Wnt7a expression has been identified as a frequent event in NSCLC, but this study is the first to suggest a mechanism of loss and to show that tobacco carcinogens may influence this loss [2].

Wnt7a plays a significant role in the maintenance of a normal epithelial phenotype in lung tissue. As such, the loss of Wnt7a could have a major impact on the development of lung cancer, especially when combined with the upregulation of an influential oncogene. This study investigates the important issue of how Wnt7a expression is lost and how its expression can be restored in an effort to treat lung cancer. Restoration of Wnt7a expression may also have potential application in other epithelial cancers or lung diseases. Therapeutic restoration of Wnt7a signaling in NSCLC could be achieved with agents that are already in clinical use, including demethylating agents and the prostacyclin analog Iloprost, which we recently identified as an activator of the Wnt7a signaling pathway [27]. Further work will be done to establish the timing of Wnt7a loss and the potential in vivo effects of treatment for *Wnt7a* methylation. The data from this study present a significant and previously undescribed step toward the development of therapeutic Wnt7a strategies for NSCLC.

## Methods

### Cell Culture, Human Tissue, and Reagents

NSCLC and Beas2B cell lines were cultured in RPMI 1640 medium supplemented with 10% fetal bovine serum at 37°C in a humidified 5% CO_2_ incubator. The HBEC (human bronchial epithelial cells) cell line was cultured in Bronchial Epithelial Basal Media at 37°C in a humidified 5% CO_2_ incubator. Cell lines were obtained from ATCC (www.atcc.org), except the HBEC cell line, which was obtained in 2009 from Dr. Robert Doebele at the University of Colorado Denver [28]. Morphology of all cells lines was monitored twice weekly and stocks of cell lines were passaged no more than ten times for use in experiments. RNA and DNA from 82 pairs of human lung tissue and adjacent normal lung tissue were obtained from the University of Colorado Cancer Center SPORE in Lung Cancer Pathology Core. NNK (Sigma) was applied at 10 uM for 2 hours in cell growth media. Cells were treated with 5-aza-2′-deoxycytidine (5 Aza) (Sigma) at 6 uM in cell growth media for 1–4 hours depending on the assay. Cigarette smoke condensate (CSC) (Murty Pharmaceuticals) was applied at a constant 1% concentration in the cell growth media for the 12 weeks.

### Knockdown studies, proliferation, 3D culture, and scratch assay

Wnt7a expression was knocked down stably in HBEC and B2B cell lines using a lentiviral shRNA system from Open Biosystems with puromycin selection. Dnmt expression was knocked down using Sure-Silencing shRNA from SABiosciences with hygromycin selection for stable expression. Transient knock down of Wnt7a was achieved with a retroviral shRNA system from Open Biosystems. Proliferation analysis was conducted in B2B cells using a 6-day cell count, where equal numbers of cells are plated in six wells and one well is counted each day. In HBEC cells, the Cell Titer Aqueous assay (Promega) was used and cells were analyzed every 24 hours for 72 hours. In 1299 cells, a clonogenicity assay was used, where 1000 cells were plated in normal growth media and stained with crystal violet for clone growth at day 4. Colonies were photographed and counted for cloning efficiency. Cloning efficiency is the number of colonies divided by the number of cells plated. Three-dimensional basement membrane cultures were established as previously described [29]. Briefly, 5,000 cells/well were grown in 2% matrigel (BD Bioscience) with EGF on a 50% matrigel base layer. For the scratch assay, cells were grown in complete growth medium until 90–100% confluent. A 3 mm space was introduced across the diameter of each plate. At time zero, cells were treated with 1 ug/ml mitomycin to inhibit cell proliferation. Cell migration was recorded at 24 hours. Images were captured using a 40× lens on a light microscope and a digital camera.

### Transfection

The reporter plasmid PPAR Response Element luciferase (PPRE) (3 µg), Fzd9 plasmid, and Wnt7a-luciferase were transfected into cells using Lipofectamine Reagent (Invitrogen; Carlsbad, CA, USA). Cells were collected, washed with PBS, and resuspended in Luciferase Reporter Lysis Buffer (Promega). The data are presented as fold-change in relative light units/milliunits of β-galactosidase and represent the average of three independent experiments. Modification of the Wnt7a luciferase was conducted with SssI, HpaII, and HhaI according to the manufacture's protocol (New England Biolabs). Transient or stable transfection of DNMT and Wnt7a shRNA was conducted using Sure-Silencing shRNA from SABiosciences or retroviral shRNA from Open Biosystems and Attractene Transfection Reagent (Qiagen).

### Quantitative PCR

RNA was extracted from cells with the AllPrep DNA/RNA Mini Kit (Qiagen) and 5 µg of RNA was converted to cDNA. PCR was conducted using SABioscience RT^2^ primers and master mix. GAPDH was used to normalize all samples. The QPCR data is presented as fold-changes in normalized mRNA levels in control vs experimental samples and are the average of at least triplicate experiments with standard error presented as error bars.

### Immunoblot analysis

Cells were harvested and protein extracts were prepared using a map kinase buffer. Proteins were detected by chemiluminescence and the ChemiDoc system (BioRad). The following antibodies were used for immunoblotting: Wnt7A (R&D Systsems), DNMT1 (Abcam) and β-actin (Abcam). β-actin was used as a loading control.

### DNA methylation analysis

DNA was isolated from cell lines and tumor samples using the AllPrep DNA/RNA Mini Kit (Qiagen) and modified using the EZ DNA Methylation-Gold kit (Zymo Research). 1 ug of DNA was used for pyrosequencing for *Wnt7a* in an assay designed and conducted by Epigendx. Data from pyrosequencing is presented as mean percent methylation, which is the mean of the percent methylation at 13 CpG sites within the CpG island in the Wnt7a promoter. The Methyl-Profiler system from SABiosciences was used to measure Wnt7a methylation in 5-aza and CSC treated cells. Data is presented as percent methylated DNA within the Wnt7a promoter.

## Supporting Information

Figure S1 **Expression of Wnt7a is not increased with DNMT3A or 3B knockdown.** QPCR was used to measure mRNA levels of DNMT3A or 3B and Wnt7a in H1299 cells with knockdown of DNMT3A or 3B. shRNA data is compared to a negative control and presented as fold-change normalized to GAPDH.
(TIF)

Table S1 **Methylation of human lung tumor tissue was analyzed using pyrosequencing.** Each tumor was analyzed at 13 CpG sites in the *Wnt7a* promoter. The percent of tumors methylated in greater than 0% of the DNA is indicated at the bottom of the table for each site. Methylation of normal lung tissue matched to the tumor tissues in Table S1 was analyzed by pyrosequencing. Each sample was analyzed at 13 CpG sites in the Wnt7a promoter. The percent of samples with methylation greater than 0% is indicated at the bottom of the table for each site.
(PDF)
