# Supplementary figures and images for: Methylation of Wnt7a Is Modulated by DNMT1 and Cigarette Smoke Condensate in Non-Small Cell Lung Cancer
Source: PLoS One. 2012 Mar 5;7(3):e32921. doi: 10.1371/journal.pone.0032921 (PMC3293913; doi:10.1371/journal.pone.0032921)

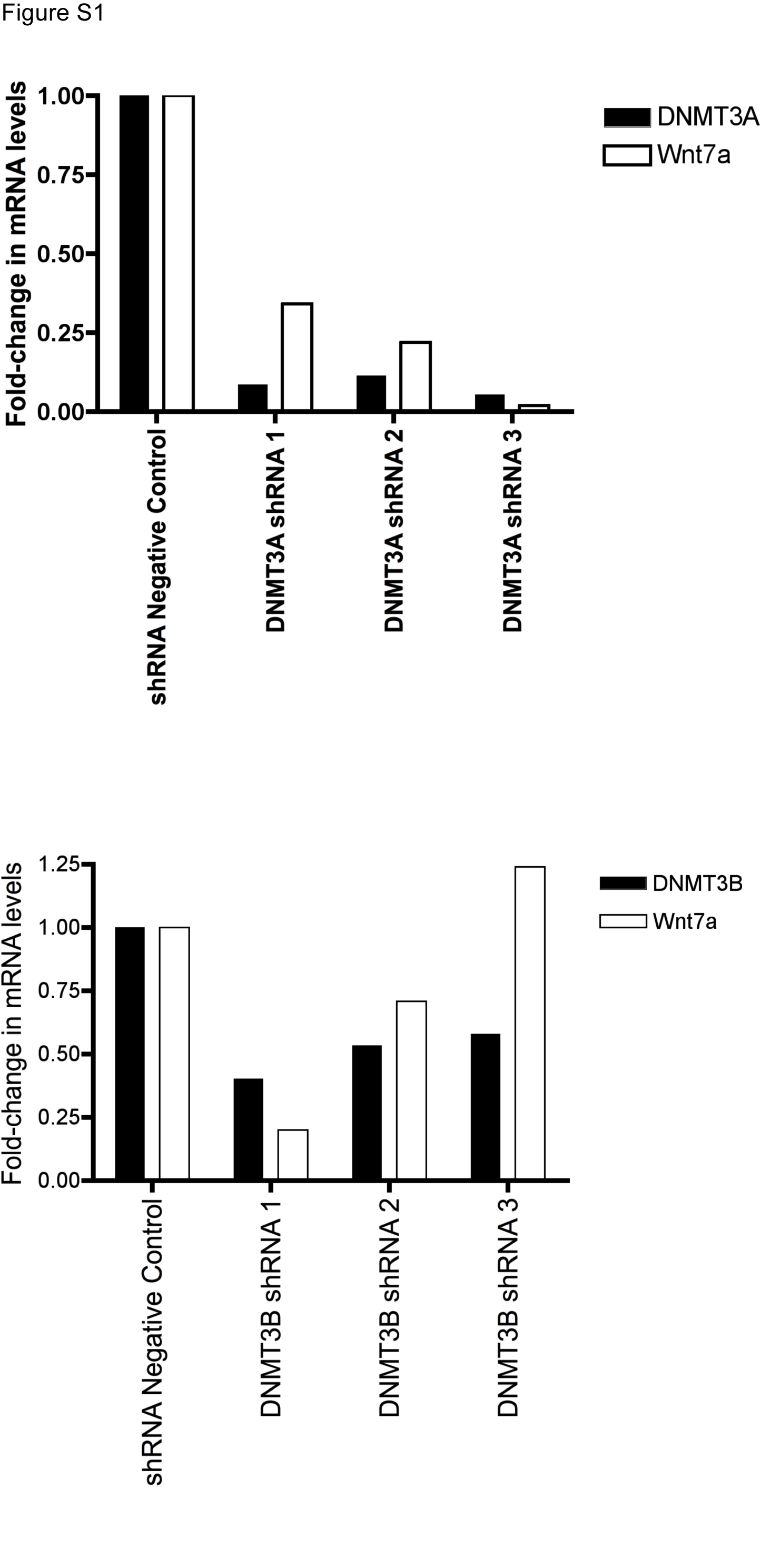

Supplement: Figure S1 — Expression of Wnt7a is not increased with DNMT3A or 3B knockdown. QPCR was used to measure mRNA levels of DNMT3A or 3B and Wnt7a in H1299 cells with knockdown of DNMT3A or 3B. shRNA data is compared to a negative control and presented as fold-change normalized to GAPDH. (TIF) [file pone.0032921.s001.tif]
