# Supplementary material for: Methylation of Wnt7a Is Modulated by DNMT1 and Cigarette Smoke Condensate in Non-Small Cell Lung Cancer
Source: PLoS One. 2012 Mar 5;7(3):e32921. doi: 10.1371/journal.pone.0032921 (PMC3293913; doi:10.1371/journal.pone.0032921)
Supplement: Table S1 — Methylation of human lung tumor tissue was analyzed using pyrosequencing. Each tumor was analyzed at 13 CpG sites in the Wnt7a promoter. The percent of tumors methylated in greater than 0% of the DNA is indicated at the bottom of the table for each site. Methylation of normal lung tissue matched to the tumor tissues in Table S1 was analyzed by pyrosequencing. Each sample was analyzed at 13 CpG sites in the Wnt7a promoter. The percent of samples with methylation greater than 0% is indicated at the bottom of the table for each site. (PDF) [file pone.0032921.s002.pdf]

Table S1

Tumor	Human Wnt7a Methylation Analysis % Methylation													
**Sample ID**	**CpG #3**	**CpG #4**	**CpG #5**	**CpG #6**	**CpG #7**	**CpG #8**	**CpG #9**	**CpG #10**	**CpG #11**	**CpG #12**	**CpG #13**	**CpG #14**	**CpG #15**	**CpG #16**
1	3.7	0.0	7.2	5.8	0.0	8.5	12.0	0.0	0.0	0.0	0.0	0.0	0.0	0.0
2	2.5	2.7	4.3	3.0	2.0	4.1	7.4	0.0	0.0	0.0	0.0	0.0	0.0	0.0
3	5.4	5.4	0.0	6.5	0.0	11.7	0.0	0.0	32.0	0.0	0.0	0.0	0.0	0.0
4	2.4	2.4	2.9	3.8	0.0	3.2	6.5	0.0	0.0	0.0	0.0	0.0	0.0	0.0
5	12.7	19.6	30.7	19.4	12.8	28.1	22.3	16.7	28.2	23.7	28.8	14.6	21.5	20.1
6	0.0	3.2	2.6	3.2	0.0	5.7	7.7	0.0	10.9	0.0	0.0	0.0	0.0	0.0
7	2.6	2.8	5.4	2.4	2.7	3.7	3.7	0.0	0.0	0.0	0.0	0.0	0.0	0.0
8	4.5	3.1	5.5	3.2	2.7	6.1	7.3	0.0	0.0	0.0	0.0	0.0	0.0	0.0
9	3.1	2.4	3.9	3.4	2.2	3.4	4.5	0.0	0.0	0.0	0.0	0.0	0.0	0.0
10	1.5	1.3	3.0	2.2	0.8	2.9	3.8	0.0	0.0	0.0	0.0	0.0	0.0	0.0
11	42.2	38.7	51.7	44.3	25.7	50.7	40.6	27.3	39.4	33.9	37.7	28.7	32.2	26.1
12	6.4	5.3	8.7	6.1	4.5	8.4	6.1	9.0	14.4	0.0	0.0	0.0	0.0	10.4
13	3.1	2.7	5.1	3.2	1.6	4.1	3.2	0.0	5.0	8.2	0.0	0.0	6.9	0.0
14	4.1	3.0	8.7	4.0	1.8	4.0	6.0	0.0	0.0	0.0	0.0	0.0	0.0	0.0
15	1.5	2.0	3.2	1.4	0.0	2.6	3.9	5.0	0.0	0.0	0.0	0.0	0.0	0.0
16	5.5	5.0	8.9	6.6	5.7	7.3	6.4	5.6	8.1	6.4	0.0	7.9	9.5	8.7
17	1.1	1.2	1.8	1.8	1.2	2.5	3.8	0.0	0.0	0.0	0.0	0.0	0.0	0.0
18	2.0	1.5	4.1	2.0	0.0	3.6	5.7	0.0	0.0	0.0	0.0	0.0	0.0	0.0
19	5.1	4.2	7.8	0.0	0.0	0.0	6.3	0.0	0.0	0.0	0.0	0.0	0.0	0.0
20	4.1	3.1	6.0	0.0	0.0	0.0	5.3	0.0	10.1	0.0	0.0	0.0	0.0	0.0
21	2.9	6.4	8.1	5.6	5.0	8.3	6.1	5.1	0.0	7.2	7.4	0.0	9.8	0.0
22	7.4	4.6	8.1	6.5	7.3	11.8	9.4	7.1	7.6	8.0	0.0	0.0	14.2	0.0
23	32.6	32.1	35.9	33.6	31.7	35.6	32.3	27.3	34.7	30.2	29.3	25.6	27.4	21.8
24	2.8	3.0	7.9	4.0	0.0	4.7	8.0	7.2	0.0	0.0	0.0	0.0	0.0	0.0
25	10.1	7.4	11.8	8.8	3.5	10.8	11.1	10.8	10.7	14.5	0.0	8.0	12.6	11.4
26	5.6	3.9	8.1	3.9	2.2	5.3	4.7	0.0	0.0	10.4	7.8	0.0	12.2	12.9
27	2.8	2.4	2.3	1.9	1.4	4.2	4.4	0.0	0.0	0.0	0.0	0.0	0.0	0.0
28	3.9	3.1	5.4	2.5	1.7	5.7	6.9	6.9	0.0	8.3	0.0	0.0	22.6	0.0
29	29.3	30.7	32.1	30.9	28.6	28.9	29.0	26.3	28.9	26.9	30.4	24.6	28.7	18.1
30	0.0	3.5	5.8	4.2	0.0	0.0	6.5	0.0	0.0	0.0	0.0	0.0	0.0	0.0
31	0.0	9.1	11.8	18.6	13.1	18.8	13.9	0.0	21.2	0.0	0.0	0.0	0.0	0.0
32	3.1	2.0	5.1	1.4	1.4	3.2	4.6	0.0	0.0	0.0	0.0	0.0	0.0	0.0
33	10.1	12.3	12.5	10.1	7.5	12.2	9.1	5.3	0.0	0.0	13.8	0.0	0.0	0.0
34	6.5	3.5	6.1	6.0	4.3	5.6	6.1	5.3	5.9	4.0	6.3	4.6	11.0	7.8

35	1.8	2.2	4.4	2.1	2.1	3.9	4.6	3.3	4.4	0.0	0.0	0.0	0.0	0.0
36	5.8	10.5	17.9	10.0	7.4	15.1	13.2	9.8	13.2	8.5	11.8	9.3	16.9	8.4
37	5.3	4.5	7.5	2.5	5.9	3.8	0.0	0.0	0.0	0.0	0.0	0.0	0.0	0.0
38	3.1	1.9	4.8	2.2	2.0	2.4	3.4	0.0	0.0	0.0	0.0	0.0	0.0	0.0
39	2.9	2.8	5.2	4.3	2.4	3.9	4.6	2.9	7.6	9.0	9.4	0.0	0.0	0.0
40	2.5	6.4	8.4	7.3	5.4	6.7	9.0	7.1	7.9	6.9	7.4	5.3	5.8	0.0
41	6.0	4.0	7.8	5.4	5.6	6.2	8.0	3.9	5.5	5.7	0.0	6.3	0.0	0.0
42	1.4	1.1	1.6	1.7	1.3	0.0	4.0	0.0	0.0	0.0	0.0	0.0	0.0	0.0
43	1.7	2.2	3.3	1.7	2.3	2.3	4.1	0.0	0.0	0.0	7.2	0.0	0.0	0.0
44	3.9	3.9	4.4	4.3	3.8	4.4	7.6	0.0	7.2	0.0	0.0	7.9	0.0	0.0
45	5.3	5.0	9.4	6.3	3.9	17.1	7.1	16.1	26.7	28.1	22.4	19.5	33.3	20.9
46	7.1	7.6	7.3	6.9	6.8	7.0	7.0	6.8	7.7	7.1	7.9	5.8	7.7	5.2
47	3.3	2.9	3.2	2.9	2.4	5.3	3.4	4.3	3.0	5.8	2.9	2.3	3.3	3.5
48	17.3	13.8	38.0	22.9	9.4	34.5	16.3	21.9	20.0	17.7	15.1	11.3	20.0	17.0
49	4.8	2.6	4.7	3.4	2.6	4.1	2.9	3.3	3.9	4.3	4.0	2.5	4.3	2.2
50	4.8	1.6	6.8	1.8	4.5	5.5	5.1	6.2	5.8	5.1	7.9	5.3	5.2	3.3
51	2.0	2.1	1.1	0.8	1.5	2.3	2.1	2.4	3.2	1.9	1.5	0.7	2.8	1.3
52	1.5	1.7	1.4	1.1	0.0	2.0	1.8	2.5	1.4	3.0	1.6	1.2	3.3	0.0
53	3.6	3.6	4.5	3.4	2.0	4.0	2.8	4.3	2.9	4.5	4.0	2.1	4.9	2.2
54	2.3	1.0	0.0	0.0	1.8	1.0	1.3	1.2	1.4	1.3	1.6	1.8	2.0	0.0
55	3.3	3.0	2.3	1.8	1.3	3.5	2.5	3.3	3.4	1.7	0.0	0.0	3.9	0.0
56	4.5	4.0	3.6	2.3	1.4	3.4	1.7	2.6	2.5	2.6	2.1	0.9	7.4	1.0
57	6.2	7.5	10.9	8.7	5.3	11.8	5.2	10.5	8.1	9.3	10.5	6.7	8.8	6.7
58	31.7	33.4	37.7	32.3	27.2	41.4	31.5	34.0	34.2	35.7	32.2	26.0	36.2	24.1
59	2.7	2.9	3.0	2.8	2.6	4.4	1.3	3.5	1.8	4.2	2.0	1.4	3.4	2.2
60	6.3	4.2	12.5	7.5	6.8	9.6	8.4	9.5	10.4	7.7	8.5	6.2	9.0	7.5
61	4.8	8.1	16.6	8.7	14.9	15.6	13.7	16.1	9.8	10.0	9.5	7.5	17.6	8.4
62	4.6	3.9	5.3	1.2	2.3	5.8	3.9	4.9	4.6	6.4	5.7	1.8	5.6	3.3
63	3.3	2.6	1.2	2.2	1.6	2.4	2.9	1.4	3.2	1.3	2.1	0.0	3.4	1.5

% methylated >0%	95	98	96	95	82	93	96	60	61	55	47	44	52	41

Normal

	Human Wnt7a Methylation Analysis													
	% Methylation													
**Sample ID**	**CpG #3**	**CpG #4**	**CpG #5**	**CpG #6**	**CpG #7**	**CpG #8**	**CpG #9**	**CpG #10**	**CpG #11**	**CpG #12**	**CpG #13**	**CpG #14**	**CpG #15**	**CpG #16**
1	0.0	0.0	6.3	0.0	0.0	0.0	14.2	0.0	0.0	0.0	0.0	0.0	0.0	0.0
2	2.6	1.5	1.3	1.1	1.4	2.7	4.7	0.0	0.0	0.0	0.0	0.0	0.0	0.0
3	1.2	1.9	0.9	1.9	1.9	5.6	0.0	0.0	0.0	0.0	0.0	0.0	0.0	0.0
4	2.1	2.4	4.1	1.9	1.5	4.2	4.7	0.0	0.0	0.0	0.0	0.0	0.0	0.0
5	4.7	5.0	5.3	0.0	0.0	5.5	8.7	8.2	0.0	0.0	23.4	0.0	0.0	0.0
6	2.6	2.7	5.2	2.6	2.8	6.3	4.3	3.9	7.5	7.8	0.0	6.4	0.0	0.0
7	1.6	1.7	4.8	2.2	0.0	3.0	3.1	0.0	8.3	0.0	0.0	0.0	10.5	0.0
8	2.7	2.2	6.3	2.6	1.8	4.6	0.0	0.0	0.0	0.0	0.0	0.0	0.0	0.0
9	3.0	3.1	7.7	2.2	1.7	6.8	7.4	8.6	0.0	7.8	0.0	9.9	0.0	0.0
10	1.5	1.5	2.7	1.9	1.3	2.1	3.1	0.0	5.7	0.0	0.0	0.0	0.0	0.0
11	2.1	1.9	3.0	1.1	1.5	3.7	3.5	5.1	8.0	0.0	0.0	0.0	0.0	0.0
12	0.0	0.0	3.5	2.7	0.0	0.0	5.3	0.0	0.0	0.0	0.0	10.8	0.0	0.0
13	2.0	2.1	5.0	1.8	0.0	0.0	5.1	0.0	0.0	0.0	0.0	0.0	0.0	0.0
14	2.1	2.9	6.2	0.0	0.0	0.0	5.3	0.0	0.0	0.0	0.0	0.0	0.0	0.0
15	3.2	3.4	6.3	3.3	0.0	3.2	5.2	0.0	0.0	0.0	0.0	0.0	0.0	0.0
16	2.0	1.8	4.1	0.0	1.0	2.8	4.3	0.0	0.0	0.0	0.0	0.0	0.0	0.0
17	1.1	2.6	0.0	0.0	0.0	2.1	5.6	0.0	0.0	0.0	0.0	0.0	0.0	0.0
18	3.4	1.2	4.9	2.5	1.5	4.9	3.8	2.5	4.6	4.0	0.0	0.0	0.0	0.0
19	3.2	1.9	5.4	2.1	2.2	2.6	3.4	4.5	0.0	0.0	0.0	0.0	0.0	0.0
20	3.8	4.0	6.9	4.3	2.5	7.0	6.2	5.2	6.5	8.4	0.0	0.0	0.0	0.0
21	2.2	1.5	3.4	1.6	0.0	2.5	3.5	3.1	0.0	4.7	0.0	0.0	0.0	0.0
22	1.3	5.1	7.1	4.8	4.7	6.2	6.5	5.4	5.6	0.0	0.0	0.0	8.3	7.8
23	0.0	0.0	0.0	0.0	0.0	0.0	5.6	0.0	0.0	0.0	0.0	0.0	0.0	0.0
24	3.0	3.2	5.5	4.2	0.0	2.3	3.7	0.0	0.0	0.0	0.0	0.0	0.0	0.0
25	4.5	4.9	11.7	5.3	3.5	5.1	6.2	0.0	0.0	0.0	0.0	0.0	0.0	0.0
26	3.5	3.4	5.9	2.3	0.0	3.8	5.3	0.0	0.0	0.0	0.0	0.0	0.0	0.0
27	2.3	3.2	4.2	1.9	1.8	3.1	3.8	0.0	0.0	0.0	0.0	0.0	0.0	0.0
28	2.4	0.0	2.1	0.0	1.2	1.7	3.9	0.0	0.0	0.0	0.0	0.0	0.0	0.0
29	1.7	1.2	2.5	2.8	1.6	1.6	3.0	2.8	3.0	0.0	0.0	0.0	0.0	0.0
30	3.5	3.3	5.0	2.5	2.0	2.5	4.6	10.3	0.0	0.0	0.0	0.0	0.0	0.0
31	0.0	0.0	12.2	0.0	0.0	0.0	6.5	0.0	0.0	0.0	8.1	0.0	0.0	0.0
32	2.4	3.7	4.3	2.7	1.5	4.0	5.0	0.0	0.0	0.0	0.0	0.0	0.0	0.0
33	1.7	2.3	4.0	1.7	1.6	3.3	0.0	0.0	0.0	0.0	0.0	0.0	0.0	0.0
34	0.0	1.2	2.2	2.3	0.0	2.9	0.0	0.0	11.1	0.0	0.0	0.0	20.1	0.0
35	1.0	1.7	1.3	1.1	1.5	2.9	2.6	0.0	0.0	0.0	0.0	0.0	0.0	0.0
36	3.6	3.0	3.7	2.8	2.0	2.4	3.9	2.9	0.0	0.0	0.0	0.0	0.0	0.0

37	2.5	3.2	6.9	3.9	0.0	3.4	4.8	0.0	0.0	0.0	0.0	0.0	0.0	0.0
38	3.5	2.4	5.8	1.8	1.8	3.3	4.2	0.0	0.0	0.0	0.0	0.0	0.0	0.0
39	3.5	0.0	0.0	0.0	0.0	0.0	15.8	0.0	0.0	0.0	0.0	0.0	0.0	0.0
40	2.0	1.3	2.1	0.0	3.0	2.2	3.4	0.0	0.0	0.0	0.0	0.0	0.0	0.0
41	1.5	0.0	2.8	1.9	1.8	4.1	5.5	0.0	0.0	0.0	10.3	0.0	0.0	0.0
42	3.3	0.8	3.6	3.1	2.6	3.6	3.3	0.0	0.0	0.0	0.0	0.0	0.0	0.0
43	1.3	2.8	4.1	5.1	1.7	2.7	5.4	6.0	0.0	0.0	0.0	0.0	0.0	0.0
44	2.4	2.0	2.2	2.0	1.9	0.0	0.0	0.0	0.0	0.0	0.0	0.0	0.0	0.0
45	1.9	1.8	2.0	2.1	1.1	2.9	1.4	2.1	1.9	4.2	3.6	2.1	5.0	3.5
46	5.7	0.9	1.0	0.0	0.0	1.4	3.3	3.1	1.0	5.6	1.9	4.1	2.4	0.7
47	1.4	1.7	0.8	0.0	0.0	1.6	1.5	4.6	1.6	1.7	2.0	0.0	4.9	1.1
48	1.1	2.6	0.0	0.0	0.0	1.2	1.6	1.7	0.0	3.9	4.8	0.0	0.0	2.8
49	3.7	1.2	2.5	2.5	1.3	5.9	2.0	3.6	1.6	3.8	3.4	2.4	3.9	1.0
50	2.5	0.0	4.1	0.0	1.4	9.5	2.6	0.0	2.8	0.0	0.0	0.0	1.8	0.0
51	1.2	4.2	2.3	0.9	5.5	3.6	4.5	11.7	3.5	4.2	3.4	2.7	2.9	2.4
52	2.9	1.3	3.4	1.9	1.8	3.6	1.5	3.7	2.5	2.8	2.2	1.5	3.5	1.6
53	2.7	2.3	2.6	0.0	0.0	2.0	1.9	0.0	2.4	2.0	1.7	1.4	2.9	1.3
54	2.3	1.1	1.6	0.0	8.8	2.5	1.4	2.2	2.1	8.4	0.0	1.9	4.5	0.0
55	2.5	1.5	2.6	1.8	1.6	3.0	2.3	3.7	3.0	2.5	4.7	1.7	2.8	1.4
56	1.8	1.8	2.7	0.8	1.3	2.5	1.7	1.9	1.4	2.7	2.9	2.0	2.3	1.2
57	1.7	3.1	3.7	2.3	1.0	3.8	1.7	4.6	2.0	4.1	3.5	1.4	4.5	1.3
58	2.2	4.3	4.7	2.5	2.5	2.7	2.0	3.2	2.5	4.0	2.7	1.7	3.9	2.1
59	1.3	3.7	1.7	2.2	2.0	5.8	2.0	2.7	0.0	3.5	2.6	0.0	2.5	1.2
60	2.9	2.4	1.7	1.4	0.0	2.6	2.4	0.0	2.1	3.2	3.1	0.0	0.0	0.0
61	0.0	3.9	0.0	0.0	0.0	0.0	0.0	0.0	0.0	0.0	7.9	0.0	0.0	0.0
62	2.3	3.5	2.2	0.0	0.0	4.0	3.1	0.0	2.9	3.7	3.9	0.0	0.0	0.0
63	2.3	3.7	2.4	2.2	1.6	4.4	2.7	3.8	2.0	3.8	2.2	0.8	2.1	1.1

% methylated >0%	90	87	92	71	63	85	90	42	39	34	31	23	28	23

Tumor/ normal	1.05	1.13	1.04	1.34	1.3	1.09	1.07	1.43	1.56	1.62	1.77	1.91	1.86	1.78
